# The ultrasound-guided selective nerve block in the upper arm: an approach of retaining the motor function in elbow

**DOI:** 10.1186/s12871-018-0584-7

**Published:** 2018-10-19

**Authors:** Weijuan Zhu, Riyong Zhou, Lulu Chen, Yuanqing Chen, Lvdan Huang, Yun Xia, Thomas J. Papadimos, Xuzhong Xu

**Affiliations:** 1 Department of Anesthesiology, The First Affiliated Hospital of Wenzhou Medical College, 2 Fuxue Road, Wenzhou City, Zhejiang Province, 325000 China; 2 Department of Anesthesiology, The Ohio State University Medical Center, Columbus, OH USA; 3 Department of Anesthesiology, The University of Toledo College of Medicine and Life Sciences, Toledo, OH Spain

**Keywords:** Ultrasonography, Nerve block, Upper extremity, Motor activity

## Abstract

**Background:**

Proximal brachial plexus blocks can lead to an extended period of motor paralysis and delay the return of motor function. This could influence patient satisfaction, and extend hospitalizations. The aim of the study is to compare a selective distal nerve block of the arm to a proximal axillary block, both ultrasound-guided, in terms of their motor block intensity of the elbow. Our hypothesis is that a selective nerve block of the arm would result in a different motor block of the elbow, compared to the axillary block.

**Methods:**

A sample size of 24 patients who were undergoing elective surgery (ASA I-III) of the wrist, hand or forearm was randomly divided into two groups: Arm Group (*n* = 12) and Axillary Group (*n* = 12). The Arm Group received ultrasound-guided block of the median, ulnar, and medial antebrachial cutaneous nerves at the level of upper-median 1/3 of the arm, and a block of the radial and musculocutaneous nerves at the level of low-median 1/3 of the arm, while the Axillary Group received ultrasound-guided axillary brachial plexus blocks. Both blocks used in combination with general anesthesia.

**Results:**

Our results demonstrated that the incidence of motor block at the elbow in the Arm Group was lower than in the Axillary Group. Compared with the Axillary Group, the duration of motor block at the elbow and the onset time of sensory block in the Arm Group were shortened. The patient satisfaction was increased in the Arm Group. There were no differences in the duration of the sensory block, the effect on postoperative analgesia, or in the duration of the motor block at the shoulder between both groups.

**Conclusion:**

Our study showed that ultrasound-guided selective nerve block in the upper arm allowed improved retention of motor function at the elbow compared to axillary block. Secondarily, the ultrasound-guided selective nerve block seemed to provide similar analgesia after surgery of the hand or forearm with an enhanced patient satisfaction.

**Trial registration:**

Chinese Clinical Trial Registry, ChiCTR-IOR-16008769. Registered 3 July 2016.

**Electronic supplementary material:**

The online version of this article (10.1186/s12871-018-0584-7) contains supplementary material, which is available to authorized users.

## Background

Most hand and wrist surgeries can be performed under ultrasound guided regional anesthesia. Interscalene, supraclavicular, axillary and infraclavicular approaches to brachial plexus blockade provide effective anesthesia for surgical procedures [1]. However, proximal brachial plexus blocks may lead to a prolonged period of motor paralysis, i.e. “dead arm.” Liebmann et al. [2] recommend that practitioners must be aware that the early return of motor function influences patient satisfaction. Chung importantly indicated that patients who recover rapidly after surgery had shortened hospitalizations and lower total costs [3]. Nonetheless there has been no technique reported thus far that provides both effective analgesia and motor function at the elbow and shoulder during upper limb surgery below the elbow.

We hypothesize that ultrasound-guided block of the median, ulnar, and medial antebrachial cutaneous nerves at the level of upper-median 1/3 of the arm, and a block of the radial and musculocutaneous nerves at the level of low-median 1/3 of the arm could improve the motor function at the elbow. We designed an approach to compare the ultrasound-guided selective nerve block of the upper arm combined general anesthesia with the axillary brachial plexus block combined general anesthesia. Our primary outcome measure was the motor function and the duration of motor blockade of the elbow and shoulder after regional anesthetic blockade. The secondary outcome measures were the anesthetic effect of the block, the onset times, the duration of the sensory blockade, the effect of postoperative analgesia, and patient satisfaction.

## Methods

The Ethics Committee at the First Affiliated Hospital of Wenzhou Medical University approved this prospective trial, and the trial was registered at the Chinese Clinical Trial Registry (ChiCTR-IOR-16008769, 2016). Written informed consent was obtained from the participants.

### Participants

We recruited 24 patients who were 18 years or older, ASA I to III, and scheduled for elective hand, wrist, and forearm surgery. Exclusion criteria were as follows: local anesthetic allergies, chronic pain, coagulopathy, infection at the planned injection site, peripheral neurologic disease, and inability to comprehend study-related procedures.

#### Procedure

The 24 patients were randomly divided into two groups: Arm Group and Axillary Group. Ultrasound-guided selective nerve block of the upper arm combined with general anesthesia were used in the Arm Group, while ultrasound-guided axillary brachial plexus block combined general anesthesia were used in the Axillary Group. On arrival in the preoperative room, all patients received standard monitoring, including noninvasive blood pressure, electrocardiogram, and pulse oximetry. A 20-gauge intravenous (IV) catheter was secured in the opposite forearm and midazolam 1 mg and fentanyl 20 μg were administered IV before nerve blockade unless contraindicated.An attending anesthesiologist with > 5 years experience and a case load of at least 200 blocks/year performed all blocks in the preoperative room using an ultrasound machine (SonoSite X-Porte, SonoSite, Bothell, WA, USA) with a linear 38 mm, 15–6 MHz probe, and 5-cm insulated block needles were used to guide a 25 ml injection of ropivacaine 0.375%. Another anesthesiologist recorded the data.

### Arm group

Ultrasound-guided selective nerve block in the upper arm combined to general anesthesia was used in the Arm Group**.** The median, ulnar and medial antebrachial cutaneous were blocked at the upper-median 1/3 of the arm; the radial nerve and the musculocutaneous nerve were blocked at the low-median 1/3 of the arm. The distance from the upper end of the humeral head to the end of olecranon was divided into three equal parts, each defined as one-third of the upper arm.

#### Upper-median 1/3 approach

**Fig. 1:**
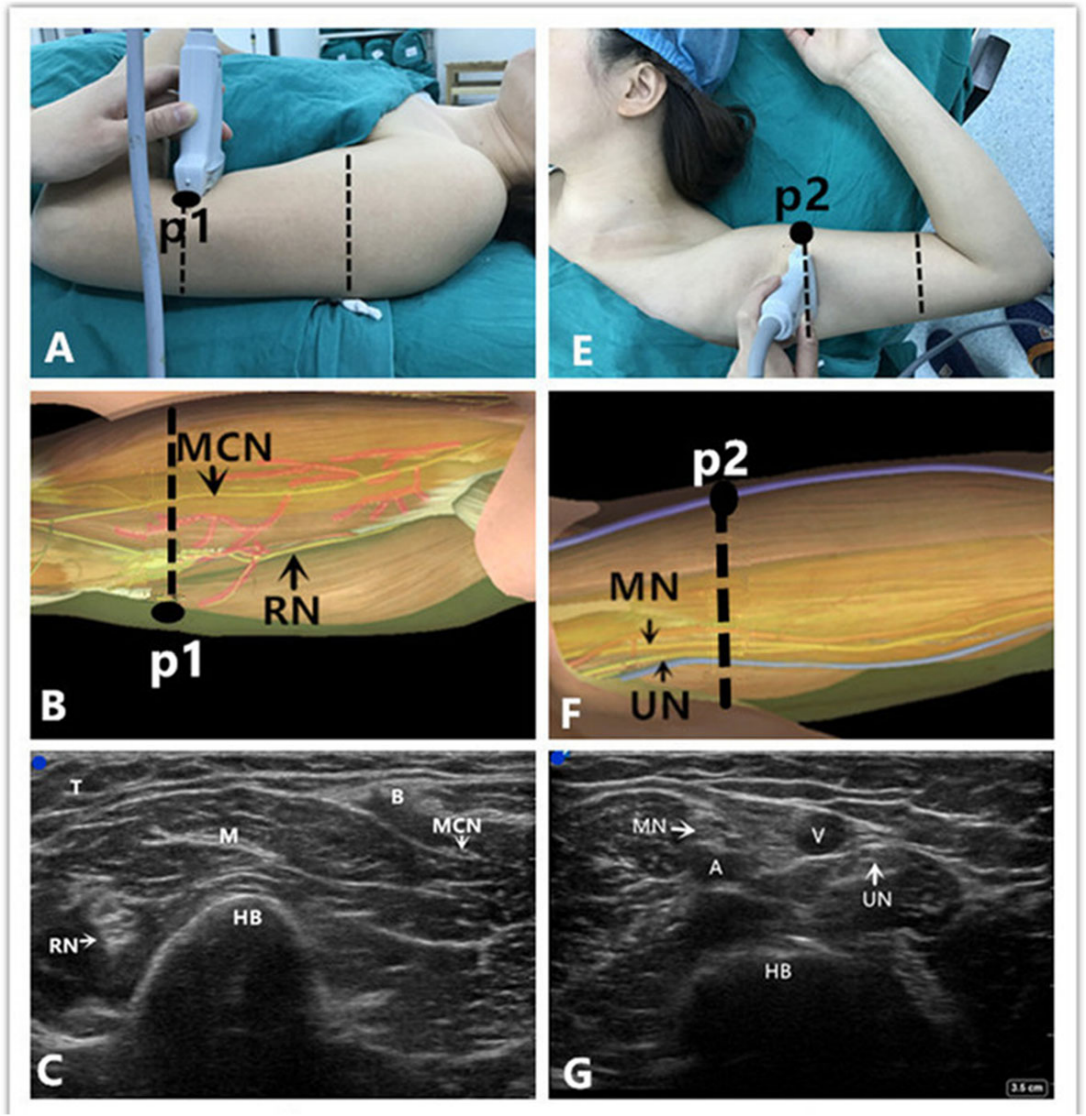
Position of patient, neuroanatomy and ultrasound imaging at the level of upper-median 1/3 and low-median 1/3 of the arm in the operating room. **a** Position of the patient, the probe, and the needle (“p1”) during block of the radial and musculocutaneous nerve. **b** Topography of the radial nerve (RN) and the musculocutaneous nerve (MCN); red = muscular branches; and the black point “p1”denotes the puncture site. The dotted line denotes the probe position. **c** Ultrasound depiction of the radial nerve (RN) and the musculocutaneous nerve (MCN). The blue point is situated at the lateral side of the probe. The RN is round with a hyperechoic structure and is located between brachialis and humerus laterally. The MCN is fusiform with a hyperechoic structure, and is located between biceps and brachialis close medially. Also note the humerus (HB), and the triceps (T), biceps (B), and brachialis (M) muscles. **e** Position of the patient, the probe, and the needle (“p2”) during block of the median, ulnar, and medial antebrachial cutaneous nerves. **f** Topography of the median nerve (MN), the ulnar nerve (UN). The black point “p2”denotes the puncture site. The dotted line denotes the probe position. **g** Ultrasound description of the median nerve (MN) and the ulnar nerve (UN). The blue point is situated at the cephalic side of the probe. The MN is between 12 and 1 o’clock in relation to the humeral artery. The UN is situated at the 3 o’clock position of the basilic vein, brachial artery (A), basilic vein (V), and humerus (HB)

The patients were placed in the supine position with one arm in abduction (90°) and externally rotated with the corresponding forearm flexed (90°) (Fig. 1e). A 15–6 MHz high-frequency linear array transducer was placed at the junction of the proximal and the middle third of the arm, perpendicular to the brachial artery. The median was between 12 and 1 o’clock in relation to the humeral artery. The ulnar and medial antebrachial cutaneous nerves were situated at the 3 o’clock position of the basilic vein (Fig. 1g). An 18-gauge, 5-cm needle was advanced along the long axis of the probe from a lateral to medial direction until its tip was positioned at the nerve. After gentle aspiration, 5 ml of ropivacaine 0.375% was injected around each nerve.

#### Low-median 1/3 approach

The arm was then abducted (Fig. 1a). The ultrasound probe was placed at the junction of the distal and the middle third of the arm. The radial nerve was identified as round with a hyperechoic structure and was located between brachialis and humerus laterally. The musculocutaneous nerve was identified as fusiform with a hyperechoic structure and was located between biceps and brachialis close medially (Fig. 1c). The needle was advanced from a lateral to medial direction using an in-plane technique until its tip was positioned at the radial nerve. After negative aspiration, 5 ml of ropivacaine 0.375% was injected around the radial nerve. The needle was then redirected toward the musculocutaneous nerve. After negative aspiration, 5 ml of ropivacaine 0.375% was injected around the musculocutaneous nerve. The radial and musculocutaneous nerves floated superiorly after local anesthetic spread and this assisted with identification.

### Axillary group

Ultrasound-guided axillary brachial plexus block combined general anesthesia was used in the Axillary Group.

**Fig. 2:**
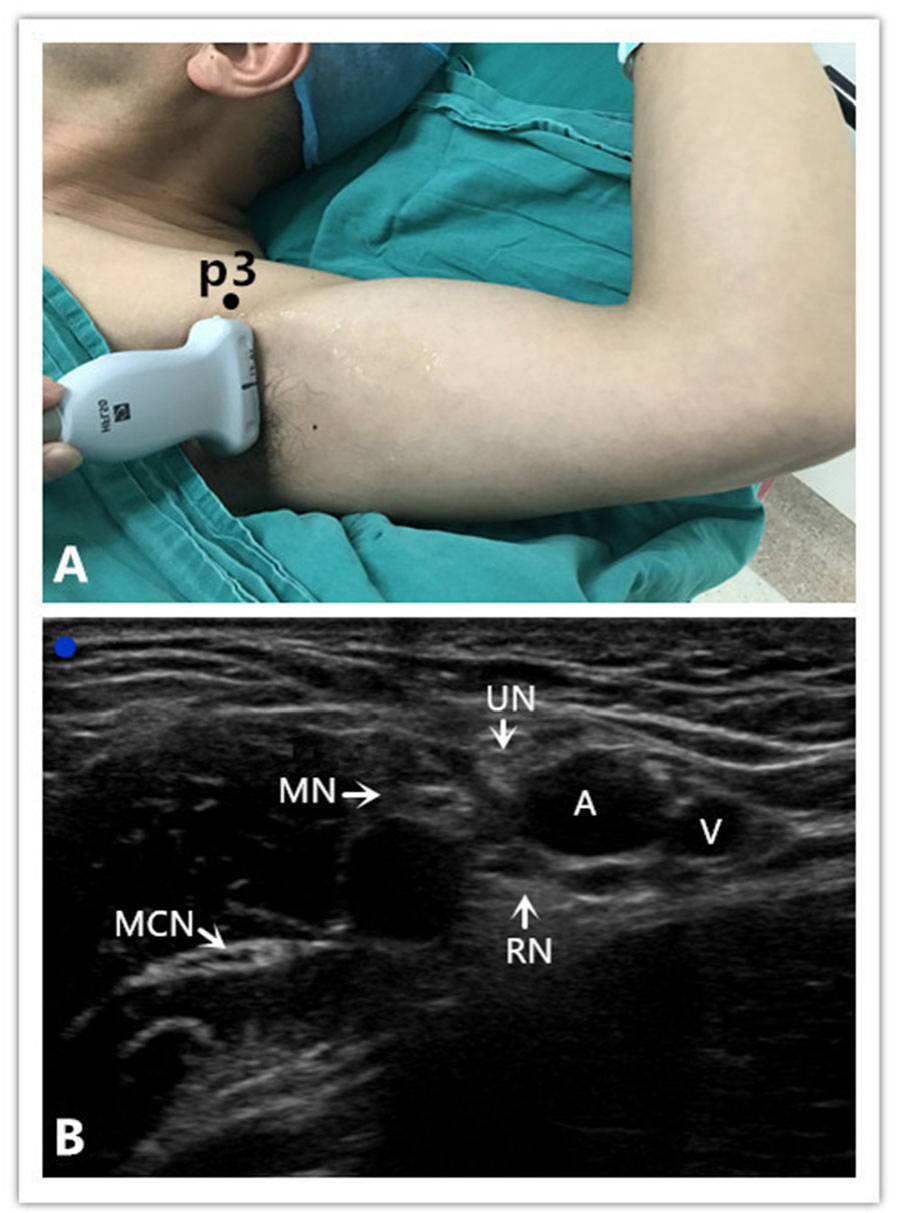
Position of patient and ultrasound imaging in the operating room when performing the axillary block. **a** Position of the patient, the probe, and the needle (“p3”) during the axillary block. **b** Ultrasound depiction of the axillary. Radial nerve (RN), musculocutaneous nerve (MCN), median nerve (MN), ulnar nerve (UN), axillary artery (A) and axillary vein (V) The blue point is situated at the lateral side of the probe

The arm is abducted to 90 degrees and the elbow flexed to 90 degrees (Fig. 2a). The ultrasound probe was placed at the lateral border of pectoralis major muscle. The pulsating axillary artery was visualized, and the probe was maneuvered to locate the individual nerves around the artery (Fig. 2b). After negative aspiration, 5 ml of ropivacaine 0.375% was injected around the median, ulnar, medial antebrachial cutaneous, radial, and musculocutaneous nerves, respectively.

Both blocks were considered successful if after 30 min there was loss of pinprick discrimination. After positioning the patient (Arm Group and Axillary Group) on the surgical table, general anesthesia was induced using propofol and fentanyl and maintained with isoflurane (to maintain a MAC between 0.8 and 1) and nitrous oxide 60% in oxygen via a laryngeal mask.

For postoperative analgesia celecoxib 200 mg po bid was administered for three days postoperatively. If VAS was > 3, Tramadol 100 mg po was administered. If thereafter the VAS was still > 3 intravenous morphine was to be administered.

### Primary measure

The primary measure of our study was the motor function and the duration of motor blockade of the elbow and shoulder. The duration of motor blockade was defined as time in minutes from the end of local anesthetic administration to the return of normal (or baseline) motor strength in the blocked extremity. Motor block was assessed for flexion and extension of the elbow, adduction and abduction of the shoulder using the following scale: 0 points for no paresis, 1 point for paresis, and 2 points for complete paralysis [4].

### Secondary measures

The secondary measures of our study were the onset and the duration of sensory blockade, patients’ satisfaction, and the postoperative analgesia. Sensory block onset times were recorded every 2 min from the end of local anesthetic administration, and were evaluated by a pinprick test [4] (using epidural plastic needle). The duration of the sensory blockade was defined as time in minutes from the end of local anesthetic administration until return of pinprick sensation or to the first report of postoperative pain at the surgical site, whichever occurred sooner. The block was identified as successful when VAS = 0 [5].

The effectiveness of postoperative analgesia was determined by the number of patients who still had a VAS > 3 after treatment with Celecoxib and Tramadol. Patients’ satisfaction scores were recorded (not satisfied at all [0] to completely satisfied [10]) [6].

### Statistical analysis

Our hypothesis is that the incidence of motor blockade with the selective distal arm block will be different from the axillary block. The sample size required for the study was calculated based on a preliminary evaluation using Power Sample Size (PASS 11) software performed in our hospital, where three patients in the Arm Group could flex and extend the elbow while one patient could not; and in the Axillary Group four patients could not flex nor extend the elbow. Thus, a calculated sample size of 6 patients per group was required to provide a statistical power of 0.90 and a type-I error of 0.05 using one-way analysis of variance. We assigned 12 patients to each group to allow for possible dropouts.

The SPSS Statistics for Windows (Version 15; IBM, Armonk, NY) was used to perform the analysis. The normality of data distribution was determined by the Shapiro-Wilk test. The non-normally distributed data (Sensory block onset, Duration time of sensory block, Duration time of motor block in elbow and shoulder, Patients’ satisfaction: see Additional file 1) are presented as a median (Q1, Q3) and analyzed with the Mann-Whitney U test. Motor block scale of elbow in Arm Group and Axillary Group are analyzed with the Chi-square test. Statistical significance was considered as *p* < 0.05.

## Results

The incidence of motor block of elbow in the Arm Group was lower than in Axillary Group [1/12vs12/12, (*P* < 0.001)] (Table 1). And compared with the Axillary Group, the duration of motor blockade in elbows of the Arm Group was shortened [0(0,0) vs. 600(495,765), *P* < 0.001]. The onset time of sensory block [Musculocutaneous nerves: 6(4.5,6) vs. 10(10,12), *P* < 0.001; Radial nerve:8(6,8) vs.10(8.5,11.5), *p* = 0.002; Ulnar nerve: 10(8,10) vs. 11(10,12), *P* = 0.007; Medial antebrachial cutaneous nerves: 10(8,10) vs. 10(10,10), *P* = 0.008; Median nerve: 10(10,10) vs. 12(10,13.5), *P* = 0.032] in the Arm Group were shortened, while the patients’ satisfaction in the Arm Group was increased [10(10,10) vs. 8(7.25,8), *P* < 0.001]. No patients with VAS > 3 were recorded in the postoperative period. There were no differences in the duration of sensory block [540(540,585) vs. 600(540,735), *P* = 0.157] and the duration of motor block of the shoulder [0(0,0) vs. 0(0,0), *P* = 1] between both groups (Table 2).

**Table 1:** Motor block scale of elbow in arm group and axillary group

	0	1	2
Arm group	11	1	0
Axillary group	0	0	12

*P* < 0.001**, 0 points for no paresis, 1 point for paresis, and 2 points for complete paralysis. *n* = 12

**Table 2:** Characteristics of arm group and axillary group (*n* = 12)

	Arm group	Axillary group	*P* value
Sensory block onset (min)			
Musculocutaneous nerves	6 (4.5,6)	10 (10,12)	*P* < 0.001**
Median nerve	10 (10,10)	12 (10,13.5)	*P* = 0.032*
Radial nerve	8 (6,8)	10 (8.5,11.5)	*P* = 0.002**
Ulnar nerve	10 (8,10)	11 (10,12)	*P* = 0.007**
Medial antebrachial cutaneous nerves	10 (8,10)	10 (10,10)	*P* = 0.008**
Duration time of sensory block (min)	540 (540,585)	600 (540,735)	*P* = 0.157
Duration time of motor block in elbow (min)	0 (0,0)	600 (495,765)	*P* < 0.001**
Duration time of motor block in shoulder (min)	0 (0,0)	0 (0,0)	*P* = 1
Patients’ satisfaction	10 (10,10)	8 (7.25,8)	*P* < 0.001**
Types of surgeries(soft-tissue/fractures/internal fixator removal)	7/ 2/ 3	7/ 1/ 4	*P* = 1

*Represent *P* < 0.05, **Represent *P* < 0.01

## Discussion

Our study showed that the selective distal ultrasound-guided block of the arm with a volume of 25 ml of ropivacaine preserves the motor function of the elbow, compared with an axillary ultrasound block by the same anesthetic agent. Compared with the Axillary Group, the duration of motor block in elbow and the onset time of sensory block in the Arm Group were shortened, while the patients’ satisfaction in the Arm Group was increased.

The ultrasound-guided selective nerve block in the upper arm assists in the retention of the motor function at the elbow whereas the proximal brachial plexus blocks do not. Proximal brachial plexus blocks as axillary may lead to a prolonged period of motor paralysis, i.e. “dead arm”. Frizelle [7] reported that the humeral canal approach to the brachial plexus (using stimulation) can provide complete sensory and motor blockade with a 90% rate of success. Carles et al. [8] reported that brachial plexus blocks at the humeral canal, using a neurostimulator, confirm the reliability and safety of this technique. However, motor function at the elbow was still impaired. Guntz et al. [9] reported that ultrasound-guided block of the brachial plexus at the humeral canal could allow the patient to recover the flexion of the forearm while providing effective post-operative analgesia. However in our study, patients could retain flexion and extension of the elbow with effective post-operative analgesia. With the increasing of ambulatory surgery in recent years, selective nerve block in the upper arm is prefer to used, because of its cost saving and reduction of medical resource requirement [3]. Our technique provided for prompt patient recovery and increased patient satisfaction, and it proved to be a basis for early patient discharge.

The radial nerve gives off branches at the level of mid brachium or proximal portion of the upper arm and innervates the triceps brachii, anconeus (extension of elbow), and the partial brachioradialis (flexion of elbow) [10]. Furthermore, the musculocutaneous nerve gives off branches at the level of the middle upper arm and innervates coracobrachiali, biceps brachii and partial brachialis muscles (flexion of elbow) [10]. The medial cutaneous nerve of the forearm gives off branches near the mid brachium; these nerves are so small that ultrasound cannot distinguish them. If an operator blocks the median, ulnar, and medial antebrachial cutaneous nerves at the low-median 1/3 of the arm, the medial antebrachial cutaneous nerves cannot be thoroughly and effectively blocked. Therefore, blocking the median, the ulnar and medial antebrachial cutaneous nerves at the upper-median 1/3 of the arm, along with a second block of the radial and the musculocutaneous nerves at the level of low-median 1/3 of the arm, provides effective analgesia for upper limb surgery below elbow, but also improves retention of the motor function at the elbow and shoulder. In our study, one patient had weak elbow extension (motor block scale = 1). Here, it is possible that the radial never gave off the triceps brachii branch near the level of the junction between the distal and the middle third of the arm.

The blocks in the two groups provided a short onset of action. English [11] reported that one of the main theories advanced for failure of axillary blockade was the existence of septa within the sheath preventing the spread of agent. Moayeri [12] reported that neural architectural differences and the varying size of adipose tissue compartments around the brachial plexus play an important role in block success. In our study, we did not need to deal with the increased adipose tissue, septa and bundles when using ultrasonography. By blocking peripheral nerves at the level of low-median 1/3 and upper-median 1/3 of the arm, where they are relative thin, allowed easy infiltration of local anesthetics. Therefore, the onset time of the sensory block was shorter and consistent with the work of Guntz [9].

Soberón [13] reported that distal peripheral nerve blocks at forearm may only be applied to wrist and hand surgery. Our approach is applicable to all hand surgeries below the elbow, while distal peripheral nerve blocks are not as appropriate or convenient. Our approach is beneficial to the promotion of ambulatory hand surgery. This study has several limitations. More distal peripheral nerve blocks have been documented to better retain the motor function at the wrist [2], but our study did not demonstrate this. And this study needed two punctures to perform the selective block. The aim of our study was observing the motor function of the elbow, while patient satisfaction was a secondary outcome. Due to the diversity of surgical procedures there may be some element of confounding in our results when measuring duration of sensory blockade. Also, as general anesthesia was used for all cases, a control group that did not receive general anesthesia was not included.

## Conclusion

In conclusion, this study showed that an ultrasound-guided block of the median, ulnar, and medial antebrachial cutaneous nerves at the level of upper-median 1/3 of the arm, and a block of the radial and musculocutaneous nerves at the level of low-median 1/3 of the arm, allowed improved retention of motor function at the elbow compared to axillary block. Secondarily, the ultrasound-guided selective nerve block seemed to provide similar analgesia after surgery of the hand or forearm with an enhanced patient satisfaction.

## Additional file

Additional file 1: Characteristics of Arm Group and Axillary Group (*n* = 12). This is the data about patients’ motor block scale and duration of motor blockade of the elbow and shoulder,the onset and the duration of sensory blockade (Musculocutaneous nerves; Radial nerve; Ulnar nerve; Medial antebrachial cutaneous nerves; Median nerve), patients’ satisfaction. (XLS 22 kb)

## Abbreviations

ASA: American Society of Anesthesiologists
IV: Intravenous
MAC: Minimal alveolar concentration
MHz: Mega Hertz
